# Pollution Confuses Pollinators. Can Scientists Retrain Them?

**DOI:** 10.1021/acscentsci.5c00779

**Published:** 2025-05-12

**Authors:** Marta Zaraska

## Abstract

As VOCs cause flower aromas to break down, bees may need to learn new tricks.

## Graphical abstract

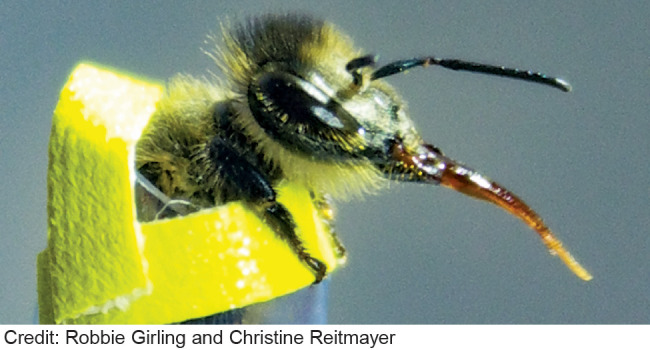

Robbie Girling gently pushes an insect through a narrow tube so that the head emerges at the other end. “The trick is making sure that your tube size is right for your bee,” he says.

Since 2010, Girling, an ecologist at the University of Reading, has trained many bees this way. While the insect is immobilized, he puffs a flowery aroma toward it and then offers a reward: a sweet solution of sucrose.

“You let them drink some of the treat so that they associate the odor with the reward,” Girling says. After three or four rounds, the bee should be trained. The next time it perceives the same aroma, it should automatically extend its proboscis expecting a treat; it is the bee equivalent of Pavlov’s dog salivating at the sound of a bell.

**Figure:**
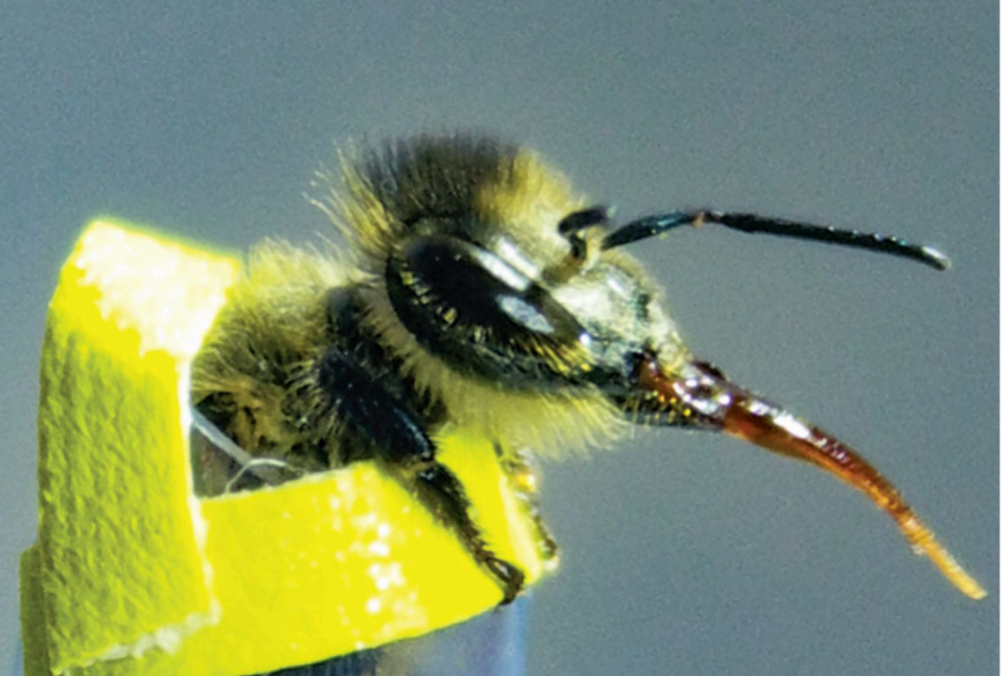
A member of Robbie Girling’s research team restrains a honeybee during scent training. The bee is being trained to extend its proboscis upon recognition of a modified floral scent. Credit: Robbie Girling/Christine Reitmayer.

Scientists like Girling train honeybees and other pollinators so the researchers can understand how the insects’ perception of flowery scents changes in polluted air. What they’ve found is troubling.

In a study reported in 2023, Girling’s team mimicked how plant odors are altered by pollutants. To do this, the researchers released plant aromas into air full of ground-level ozone—a gas that can occur naturally but is primarily formed when human-made pollutants react with other airborne chemicals in sunlight. In such a scenario, most honeybees couldn’t recognize the distorted scents at all.

Other research shows that air pollution breaks down the molecules that make up the aroma of flowers. Ozone, for instance, can cause lavender to develop sour notes. Eucalyptus, meanwhile, might start smelling like orchids. That might seem nice to a human, but for an insect who depends on finding eucalyptus for survival, it could pose an existential problem.

A 2024 meta-analysis by Girling and his colleagues found that air pollution spells trouble for beneficial invertebrates, including pollinators and parasitoids—animals like parasitic wasps—which are natural enemies of crop pests. Across 120 publications from 19 countries, ozone pollution decreased performance of beneficial invertebrates by more than 31%, while nitrogen oxides (NO_*x*_) decreased it by 24%. The results suggest that pollution’s negative effects most likely have to do with insects not being able to find food, such as honeybees having a harder time finding nectar.

This could mean less pollination. Given that three-quarters of leading food crops depend on animal pollination, air pollution “can actually decrease crop production,” says Jeffrey Riffell, an ecologist at the University of Washington who was not involved in the meta-analysis.

Disturbing these tiny creatures can have profound consequences on human well-being. This is why researchers are trying to better understand how increasing levels of pollution have led to a communication breakdown between pollinators and plants. Through hands-on work with insects and an analysis of bouquets of floral molecules, they are starting to identify which fragrant compounds are most essential to a functional ecosystem and which ones are most at risk.

## When molecules meet midair

To locate flower patches over long distances, insect pollinators tend to rely on their sense of smell, which, Riffell says, is much better than their vision. “Some of them can actually smell from a kilometer away,” he says. Like humans, insects use a combination of chemicals to recognize an aroma of flowers, either noting the specific compounds or judging the mixture by its proportions. “When we smell a rose, we are actually smelling hundreds of chemicals all at the same time, and we almost instantaneously recognize it as a rose,” Riffell says.

Plants release thousands of volatile organic compounds (VOCs). Some are emitted by leaves, and some by the roots. Hundreds, if not thousands, are emitted by flowers: the latest tally stands at about 1,700 floral volatiles known to science, and new ones are constantly being discovered.

Scientists have found that VOCs emitted by flowers are primarily benzenoids, fatty acid derivatives, and most notably, terpenes and terpenoids, including sweet-smelling linalool, citrusy limonene, and spicy myrcene. “I like to think of them as beacons. They’re what the flower uses to call insects,” says Ben Langford, an atmospheric scientist at the UK Centre for Ecology and Hydrology.

**Figure:**
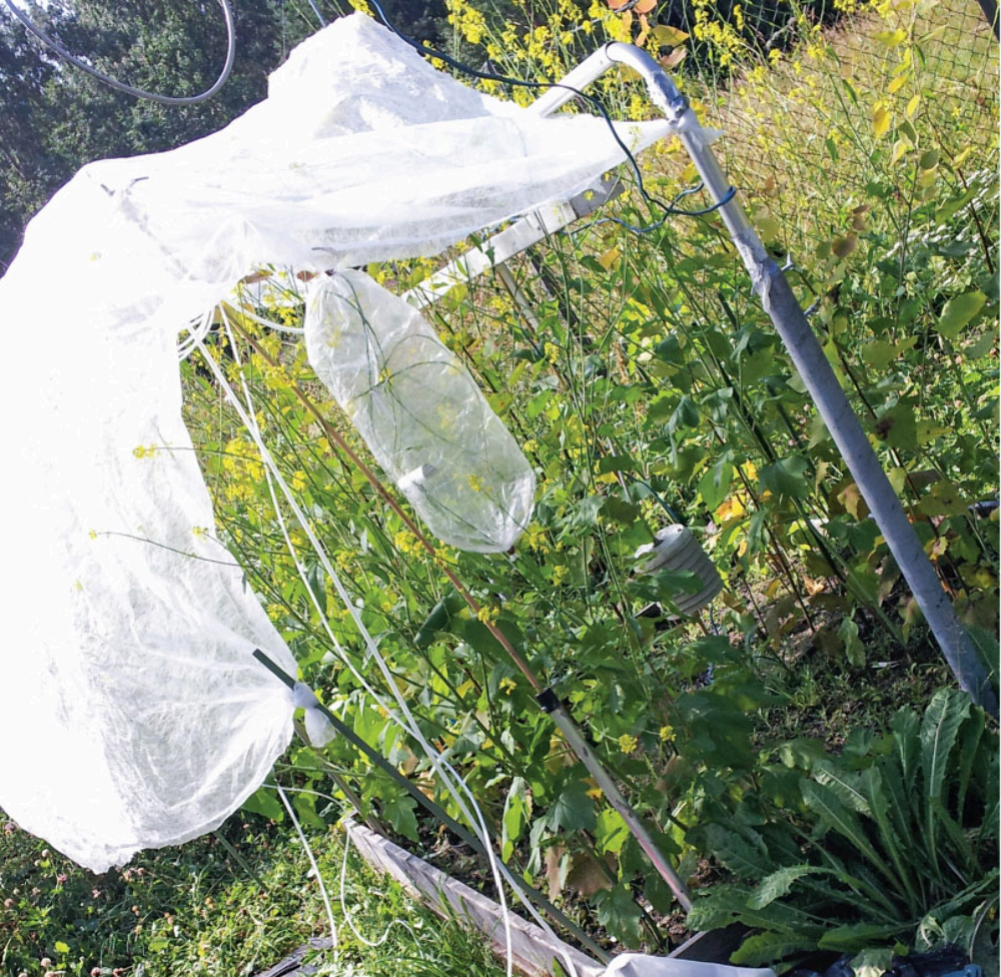
James Blande’s research team in Kuopio, Finland, placed bags over flowers, like this black mustard, for an experiment in 2011. The researchers sampled the volatile chemicals the flowers released and analyzed them using gas chromatography and mass spectrometry. Credit: James Blande.

But because of their carbon–carbon double bonds, terpenes are quite reactive with certain pollutants in the air, such as ozone and nitrate radicals (^•^NO_3_). In the preindustrial past, this made adaptive sense. When flowers bloomed, they sent out powerful but short-lived compounds that could guide insects right to them. As those molecules drifted farther from their flowers, the natural ozone and radicals in the air would degrade them. Without that natural degradation, aroma compounds would have been picked up so far from their source that they would be useless to insects—imagine smelling freshly baked cookies when they’re in fact 100 km away.

Ozone in the air has its own regulation system. In a natural state, “ozone is formed and destroyed in this circular motion,” Langford says. Ground-level ozone is formed primarily from reactions between NO_*x*_ and VOCs, both natural and human-made ones, in the presence of sunlight. Nitric oxide also reacts with ozone, leaving behind oxygen and nitrogen dioxide and reducing ozone's overall concentration.

**Figure:**
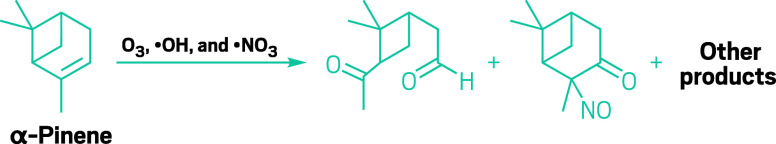
Volatile chemicals such as α-pinene can react in myriad ways with ozone and radicals in the air.

But today’s levels of human-created pollution have disturbed that balance, wreaking havoc on insect navigation. We’ve added to the mixture large doses of anthropogenic VOCs emitted from chemical plants, gasoline pumps, and auto body shops. Such VOCs drive up the levels of ground-level ozone. Between preindustrial times and the beginning of the 21st century, ground-level ozone concentrations rose from about 5–15 parts per billion (ppb) to an annual average of 20–45 ppb over the midlatitudes of the Northern Hemisphere. On bad days in some regions, such as during summer heat waves in Athens, Greece, ozone levels can exceed 200 ppb.

That extra ozone is now degrading many floral aroma molecules more quickly. A 2024 study by Girling, Langford, and their colleagues showed that terpenes’ double bonds are very vulnerable to airborne ozonolysis. The double bond is “the area of the molecule that’s the easiest to crack open,” Langford says. A chain of reactions follows, leading to the production of carbonyls, carboxylic acids, and minor byproducts such as alcohols and esters. All these introduce “more noise into the system” and confuse insects, Langford says.

## Probing pollinator problems

Researchers are now working to figure out what these chemical changes mean for pollinators, plants, and ecosystems. Certain floral VOCs, for example, are particularly sensitive to ozonolysis: they might vanish in minutes, while others linger for hours. A 2016 modeling study showed that while most reactive compounds can be found only directly above floral patches in ozone-polluted air, the least reactive ones can travel a quarter mile downwind.

As a result, a flower’s overall scent can change a lot with distance, making it harder for pollinators to recognize the signature aroma of a particular plant. (Imagine if a few notes were to shift in the scent of an orange; it might start smelling like a lemon instead.) Bumblebees, for one, no longer recognize the scent of black mustard, a plant with small yellow flowers resembling canola, after the odor molecules pass through ozone.

**Figure:**
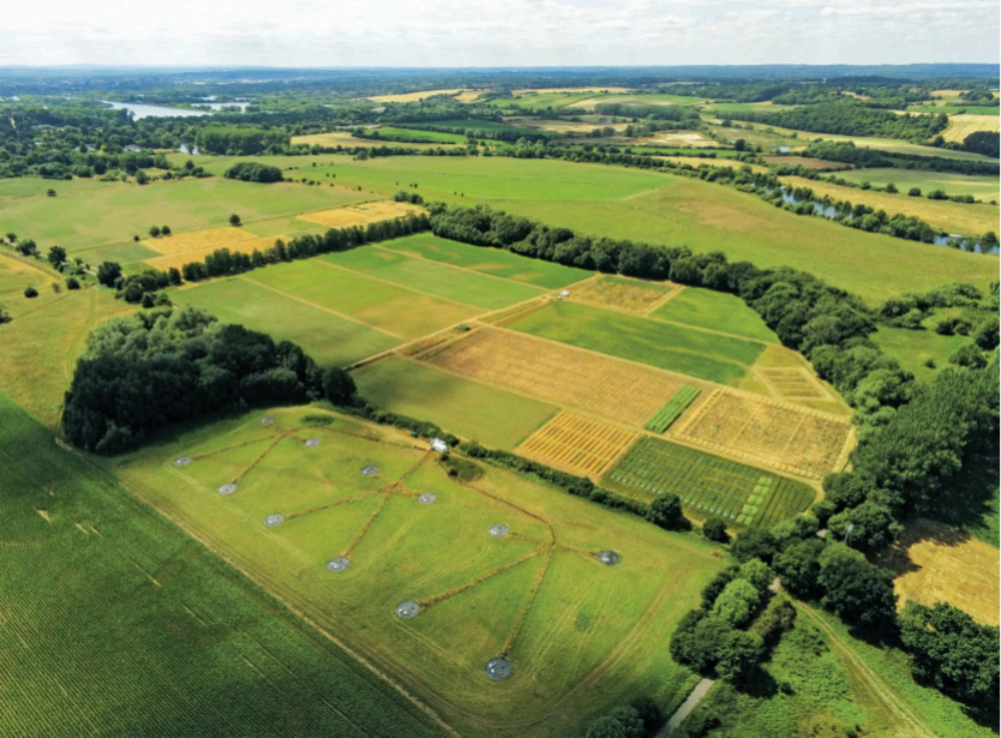
At the Free-Air Diesel and Ozone Enrichment facility at the University of Reading’s Sonning Farm, Robbie Girling and his colleagues conduct field experiments investigating how ozone pollution translates into fewer pollinators visiting flowers. Credit: University of Reading.

In one study, researchers at the Spanish National Research Council and the University of Eastern Finland gave insects a choice between two paper flowers cut to resemble black mustard and placed at opposing ends of a cylindrical arena. Tubes placed behind the flowers pumped either clean air, floral VOCs, or floral VOCs that were degraded by ozone.

The researchers released bumblebees one by one into the arena to check which paper flower they would choose. They observed that the pollinators were clearly drawn to the bloom associated with the original floral scent, but they could not pick between flowers associated with clean air and air with VOCs that ozone had heavily degraded—apparently, neither appealed to them.

In another study to test how flowery aromas change over much larger distances, Langford, Girling, and their colleagues used a 20 m long research tunnel that resembles an oversize shipping container. The scientists reconstructed aromas of flowers in the laboratory (real plants would wilt too fast, Langford says) and pumped these floral VOCs into the tunnel alongside ozone, sampling the air in dozens of locations.

**Figure:**
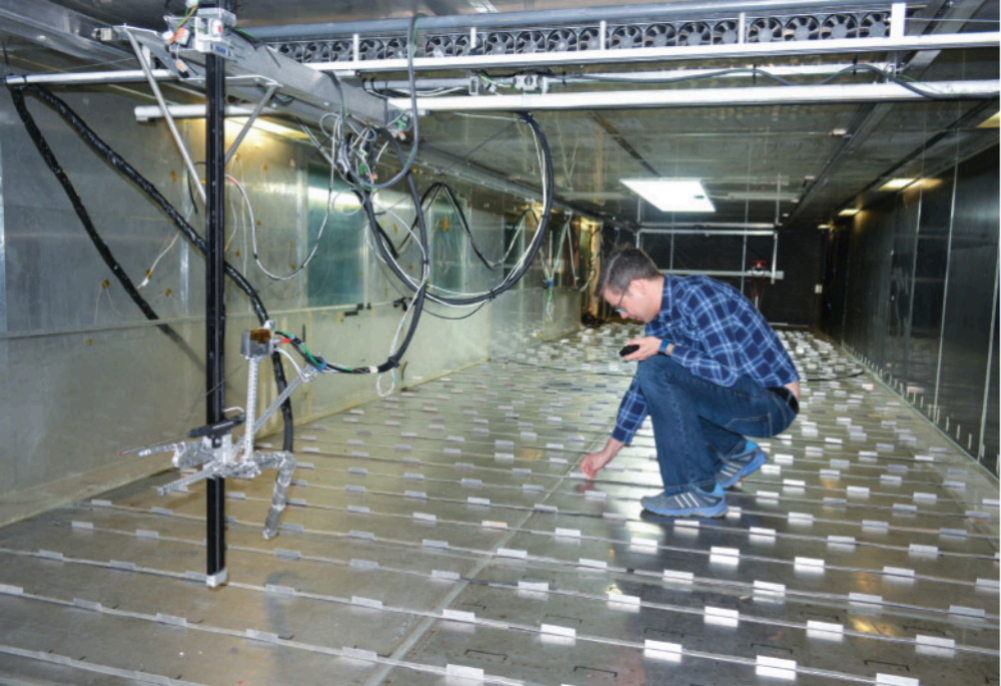
Robbie Girling sets up a wind tunnel in which he and Ben Langford conduct experiments on how ozone degrades floral scents. Credit: Robbie Girling/Neil Mullinger.

Ozone, they found, not only degraded the floral compounds but also changed the “shapes” of the scents’ plumes. Flowery aromas normally behave like cigarette smoke, Girling says, curling through the air in filaments, some thicker, some thinner. Insects catch a whiff of these wisps and fly upwind, following the tendrils of VOCs to their source, he says. But ozone narrows both the individual filaments and the plume as a whole, making it potentially harder for insects to navigate to its source.

As a result, in real-world conditions, pollinators ignore flower patches polluted by ozone and NO_*x*_. A 2025 field study showed that plots of black mustard were visited by 37% fewer pollinators if researchers pumped ozone over them, compared with flowers growing in unpolluted sites. When the pollution came from both ozone and diesel in concentrations commonly reported next to major UK roads, the number of pollinators dropped by almost half compared with that in control conditions.

While ozone degrades the scents of flowers during the day, at nighttime, it is mostly ^•^NO_3_—a compound that forms in the air from fossil fuel pollution—that causes trouble. “It doesn’t exist during the daytime because it undergoes rapid photolysis in daylight,” Girling says. Meanwhile, he says, “if you are a nocturnal insect, odor is everything.”

Riffell and his colleagues studied how ^•^NO_3_ degrades the scent of pale evening primrose and what that means for hawkmoths, nighttime pollinators that hover and sip nectar midair like hummingbirds. They discovered that ^•^NO_3_ reduces the levels of pine-smelling β-pinene in the scent of primrose by 84% and reduces woody β-ocimene by 67%. In the field, such changes mean fewer hawkmoths visit ^•^NO_3_-polluted flowers. “This causes the plants to have really decreased fitness,” Riffell says.

## Old insects, new tricks

Yet there may be hope for pollinators and crops in a more polluted world. Some studies suggest that certain pollinators can learn scents that have been altered by air pollution.

Getting bugs to crave certain flowers has a long legacy. To improve food production, farmers have been training honeybees since well before World War II.

The farmers would offer bees sugar syrup scented with the flowers of the target crop, placing the mixture inside a beehive. This way, the bees would learn to pollinate what the farmers wanted, and not, say, random wildflowers. Farmers today successfully conduct similar scent training.

Taking cues from that technique, researchers are trying to rewire the insects to seek out scents degraded by ozone, in a way similar to what Girling does with his bees. In a 2020 study, an international team managed to train hawkmoths using paper flowers, modeled on jasmine tobacco, that emitted ozone-altered scents. The fake flowers were infused with sucrose as a reward for the insects. After such training, the pollinators were more eager than before to extend their proboscis in response to pollution-degraded aromas.

A challenge is that floral scents change constantly in polluted air: with distance from the source and with daily pollution patterns.

What’s more, not all pollinators can be trained. “Some species of insects rely on learning to find resources, and others do not,” says Magali Proffit, an ecologist at the French National Center for Scientific Research (CNRS).

Her recent study on fig wasps, the sole pollinators of Mediterranean fig trees, found that while these insects stop recognizing the scent of figs in ozone-polluted air, they have no opportunity to learn and apply their new knowledge: “They live less than 24 h, and the main aim in their life is to find a fig,” Proffit says. As such, she says, the ability to learn has not been prioritized in the evolution of this species.

Another option would be to breed or genetically engineer plants to produce stronger scents. These strategies have already been applied in several crops, such as tomatoes and carnations, though not to help out pollinators. (Fragrant flowers and fruits tend to appeal more to humans too.)

James Blande, an ecologist at the University of Eastern Finland, says these are “fascinating ideas,” but he believes there are better, simpler options than training bees or engineering crops. “The most realistic way to address this is through how we prepare our agricultural environments,” he says. Basically, pollinators need flowers to be easy to find.

“The countryside now, if you look at it, it is very green—but there are not a lot of flowers. We tend to have these large expanses of monocultures,” Langford says. As such, pollinators are “becoming a lot more reliant on their sense of smell to find where the patches of flowers are among all the green,” he says. The solution would be to plant more pollinator-friendly flowers among crops, drawing the insects in.

Across the globe, pollinator populations are in “huge decline,” Langford says. They are threatened by pesticides, by climate change, by emerging diseases. In North America, the number of bumblebees has dropped by nearly 50% since 1974, according to the US Fish and Wildlife Service. And the European Commission reports that 1 in 10 bee and butterfly species in Europe is threatened with extinction.

“Air pollution is definitely not the sole reason behind that,” Langford says, but it is “an added stress on an already very stressed system.”

*Marta Zaraska is a* freelance *contributor to* Chemical & Engineering News, *the independent news outlet of the American Chemical Society*.

